# Cochlear histological changes after noise exposure: effects of melatonin and resveratrol in an experimental model

**DOI:** 10.1016/j.bjorl.2026.101930

**Published:** 2026-10-14

**Authors:** Gabriela Guenther Ribeiro Novanta, Ana Carolina Odorizzi Zica, Maria Luiza Queiroz Sampaio, Andressa Alves Caram, Selma Aparecida Souza Kückelhaus, Lucieny Silva Martins Serra, Andre Luiz Lopes Sampaio

**Affiliations:** a Faculdade de Medicina da Universidade de Brasília, Laboratory of Otorhinolaryngology Teaching and Research (LEO), Campus Darcy Ribeiro, Asa Norte, Brasília, DF, Brazil; b Faculdade de Medicina da Universidade de Brasília, Research Center for Applied Morphology and Immunology (NuPMIA), Campus Darcy Ribeiro, Asa Norte, Brasília, DF, Brazil

**Keywords:** Hearing loss, Melatonin, Resveratrol, Noise, Rats

## Abstract

**Objective:**

To assess cochlear histological changes and the otoprotective effects of melatonin and resveratrol in Wistar rats exposed to high sound pressure levels.

**Methods:**

This was a prospective, longitudinal, in vivo experimental study with heterogeneous male Wistar rats. The animals were divided into groups and treated with or without 10 mg/day of melatonin or resveratrol administered intraperitoneally for 14 days. They were then exposed to or not exposed to white noise (4 kHz; 100 ± 3 dB) for 8 h daily for 10-days. Histological analyses were performed on the spiral ganglion, spiral limbus, spiral ligament, and stria vascularis on days 15 (D15) and 30 (D30), after euthanasia. The data were analyzed by ANOVA.

**Results:**

The animals in the control group had a higher number of cells in the analyzed cochlear structures than the noise-exposed group. Treatment with melatonin or resveratrol prevented the noise-induced reduction in the number of neurons in the spiral ganglion, maintaining cell counts similar to those in the control group. Resveratrol was also effective in preserving the spiral ligament.

**Conclusion:**

The findings suggest that the preventive administration of antioxidants may be an effective strategy in mitigating noise-induced hearing loss, reinforcing the therapeutic potential of these compounds in preventing hearing impairment.

**Level of evidence:**

V.

## Highlights

• Melatonin and resveratrol show otoprotective effects in Wistar rats.
• Melatonin and resveratrol preserved spiral ganglion neurons after noise exposure.
• Findings highlight potential hearing protection against noise damage.

## Introduction

Noise-Induced Hearing Loss (NIHL) or Hearing loss Induced by High Sound pressure Levels (HISHL) remains one of the most widespread and prevalent occupational health problems worldwide. According to recent global estimates by the World Health Organization and the International Labour Organization, occupational noise exposure accounts for several million Years Lived with Disability (YLDs), remaining a primary contributor to the global burden of work-related diseases.^1^

Noise exposure induces excessive glutamate release from inner hair cells, causing synaptic excitotoxicity and reversible dendritic edema (auditory fatigue). Simultaneously, oxidative stress generated by mitochondrial hyperactivity produces Reactive Oxygen Species (ROS) that damage hair cells, contributing to permanent hearing loss.^2^^,^^3^ The effects of noise can also affect hair cell synapses and neurons, even when the latter remain alive and recover normal function. Studies suggest that the loss of synapses and afferent terminals due to noise exposure is rapid and permanent, while the loss of spiral ganglion neurons is comparatively slow.^4^^,^^5^ This synaptic and neural loss may extrapolate the functional consequences of noise exposure by decreasing the ability of the VIII nerve to encode auditory signals faithfully, with or without loss of threshold sensitivity.^6^^,^^7^

Given the difficulty in reducing sound pressure levels in some work or social environments, the use of antioxidant substances, including melatonin and resveratrol, in the prevention of hearing loss has been widely studied in recent years.

Studies show that both compounds have complementary pharmacokinetic profiles for otoprotective therapies. Melatonin stands out for its ability to concentrate in mitochondria, the site of free radical generation. Thus, it has a positional advantage within cells, which improves its effectiveness in eliminating toxic radicals and reducing associated oxidative stress.^8^, ^9^, ^10^ In turn, resveratrol’s low molecular weight and lipophilicity facilitate its diffusion to deep cellular compartments, such as the inner ear, in addition to its diverse biological properties, including antioxidant, anti-inflammatory, anti-aggregant, anti-atherogenic, chemoprevention, immunomodulation, and anti-aging effects.^10^, ^11^, ^12^, ^13^, ^14^

This study aimed to evaluate the effect of melatonin and resveratrol through a morphological and morphometric analysis of the cochlear structures of Wistar rats exposed to high sound pressure levels.

## Methods

### Type of study

This was an experimental, prospective, comparative, longitudinal study with heterogeneous male Wistar rats aged ∼40 days. The study was approved by the Animal Research Ethics Committee of the Universidade de Brasília (No. 23106.001211/2020-39) and conducted in accordance with the guidelines of the Conselho Nacional de Controle de Experimentação Animal - CONCEA.

### Study and treatment groups

1) Control (C; n = 4), animals treated with saline and not exposed to noise; 2) Noise (No; n = 8), animals treated with saline and exposed to noise; 3) Resveratrol Noise (Re/No; n = 8), animals treated with resveratrol and exposed to noise; 4) Melatonin Noise (Me/No; n = 8), animals treated with melatonin and exposed to noise.

### Drug administration

The drugs, resveratrol (Changsha Huir Biological-Tech, China) or melatonin (Changsha Huir Biological-Tech, China), were administered intraperitoneally to the animals in the test groups (Re/No and Me/No) (10 mg/kg/day) for 14 consecutive days, starting 4 days before noise exposure. The animals in the C and No groups received 0.5 mL/saline/day intraperitoneally during the same period.

### Noise exposure

The animals were exposed to white noise centered at 4000 Hz (3564–4490 Hz) and 100 dB SPL for 8 h daily for 10 consecutive days. The noise was monitored by sampling every minute using type S temporal weighting and type A frequency weighting. The recorded values included a minimum intensity (valley) of 100.6 dB, a peak of 103.0 dB, and dominant frequencies at 3150 Hz and 5000 Hz. The average intensities in the frequency bands were 86.9 dB at 3150 Hz, 100.6 dB at 4000 Hz, and 90.7 dB at 5000 Hz.

### Functional and electrophysiological assessment of hearing

The animals were euthanized at two time points: on D15, after the end of noise exposure, and on D30, after 15 days of rest. The animals were anesthetized with ketamine hydrochloride (65 mg/kg; 50 mg/mL) and xylazine (6.5 mg/kg; 20 mg/mL) and placed in a chamber with a 40% CO_2_ concentration, resulting in death by excessive central nervous system depression and hypoxia due to oxygen displacement during alveolar gas exchange. After euthanasia, the animals were perfused through the heart with saline to remove blood, and then a 10% paraformaldehyde solution was injected to fix the tissues. The heads were then excised for histological studies. The anatomical specimens were processed according to the standard protocol for routine histological examination, immersing them in solutions with increasing concentrations of alcohol for dehydration (70%, 80%, 90%, and 3 × 99.8%) for up to 1 h each, followed by three xylene baths for up to 1 -h each for fat removal (diaphanization); then, they were added to Paraplast® synthetic resin (Sigma Aldrich).

The specimens were embedded in two anatomical planes: transverse and sagittal. Microtomy began on the surface of the bullae (anterior and lateral surfaces), and histological sections were made with a thickness of 6 μm in both planes. The slides were stained with hematoxylin and eosin. After staining, the images were captured with the AperioScanScope® equipment and evaluated in the ImageScopeversion 11.2.0.780 program (Aperio Technologies Inc, Vista, CA, USA) by a blinded examiner (20×).

### Analysis of histological data

A single-blind evaluation was performed to analyze the photomicrographs of the slides, using the criterion of viable cells per area. Viable cells present in each photomicrograph of the material were quantified to analyze cell density, fractionated by the selected area: 80 μm^2^ for analysis of the spiral ganglion, 50 μm^2^ for analysis of the limbus, 100 × 25 μm for analysis of the spiral ligament, and 100 linear μm for analysis of the stria vascularis.

### Statistical analysis

The normality of the variables was analyzed using the Kolmogorov-Smirnov test, and the homogeneity of variances was analyzed using the Bartlett test. The ANOVA test was used to compare the groups, and differences with p < 0.05 were considered statistically significant. The analyses were performed using the Prism® 5 software package (GraphPad, USA, 2005).

## Results

The study groups were divided into two based on the timing of euthanasia. Thus, animals euthanized on D15, the day after the end of noise exposure, were designated D15, and those euthanized on D30 (remaining acoustically rested for 15 days after the end of noise exposure) were designated D30. Animals in the C group were euthanized only on D30.

### Spiral ganglion

The comparison of results of the median total viable ganglion neurons of group C with the noise-exposed groups (Me/No, Re/No, and No) on D15 found a statistical difference in all groups (ANOVA p = 0.0018). However, the same comparison on D30 found that only the No group maintained a statistical difference (ANOVA p = 0.0003). This difference was also observed when comparing the No group with the Re/No and Me/No groups (Fig. 1).

**Fig. 1:**
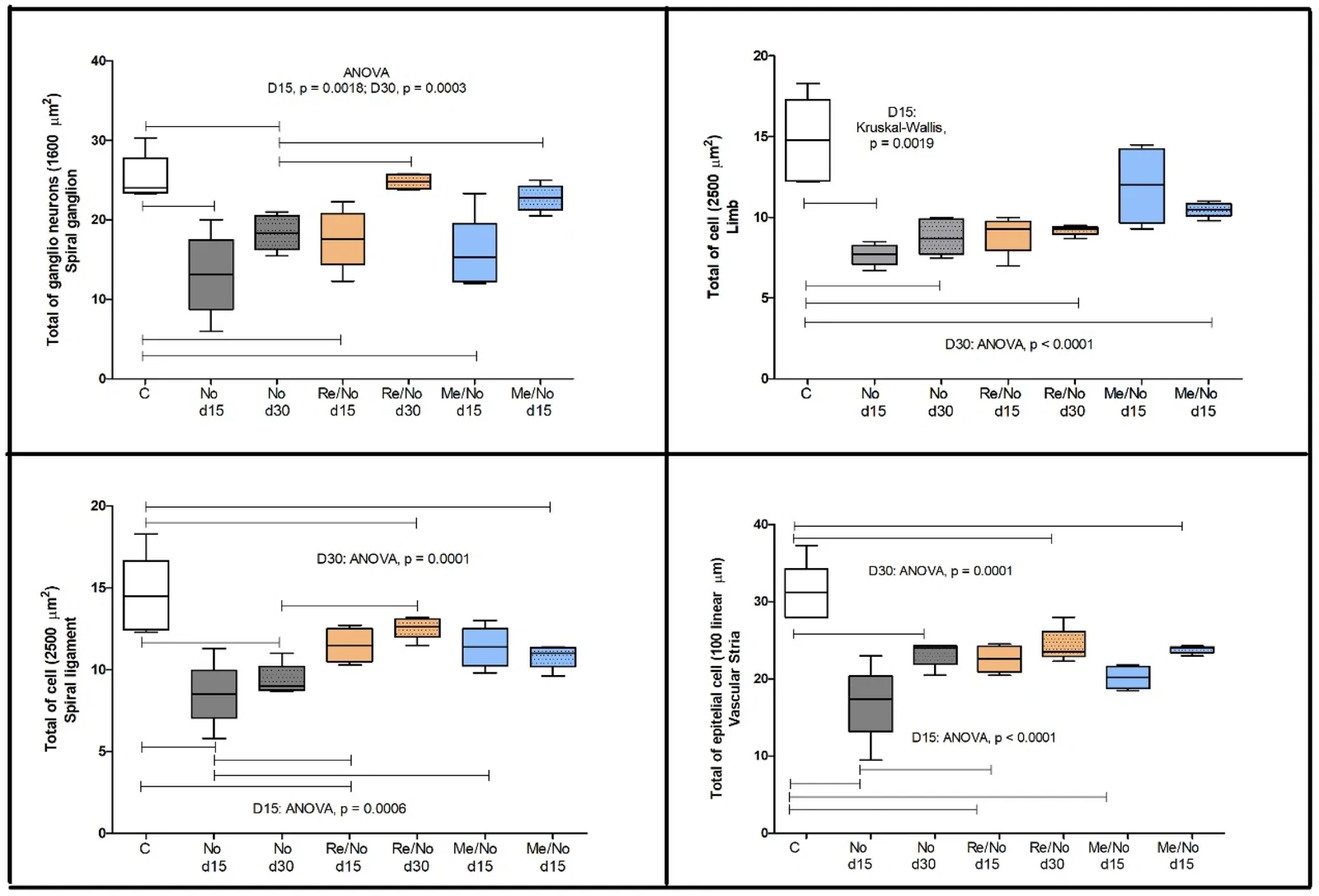
Total cells/area obtained from the cochlear tissues of Wistar rats: spiral ganglion, spiral limbus, spiral ligament, and stria vascularis. The groups of animals were exposed to white noise for 15 or 30 days and/or treated with 10 mg/kg/day of resveratrol or melatonin for 15 or 30 days. Medians, quartiles, maximum and minimum values, and extreme points are shown.

Fig. 2 shows the photomicrograph of the spiral ganglion of animals in the C and exposed No, Me/No, and Re/No groups. By counting neurons in a defined area, it can be observed that cell density was reduced in animals euthanized immediately after exposure. In animals treated with melatonin and resveratrol euthanized after acoustic rest (D30), a density similar to that of animals in group C was observed.

**Fig. 2:**
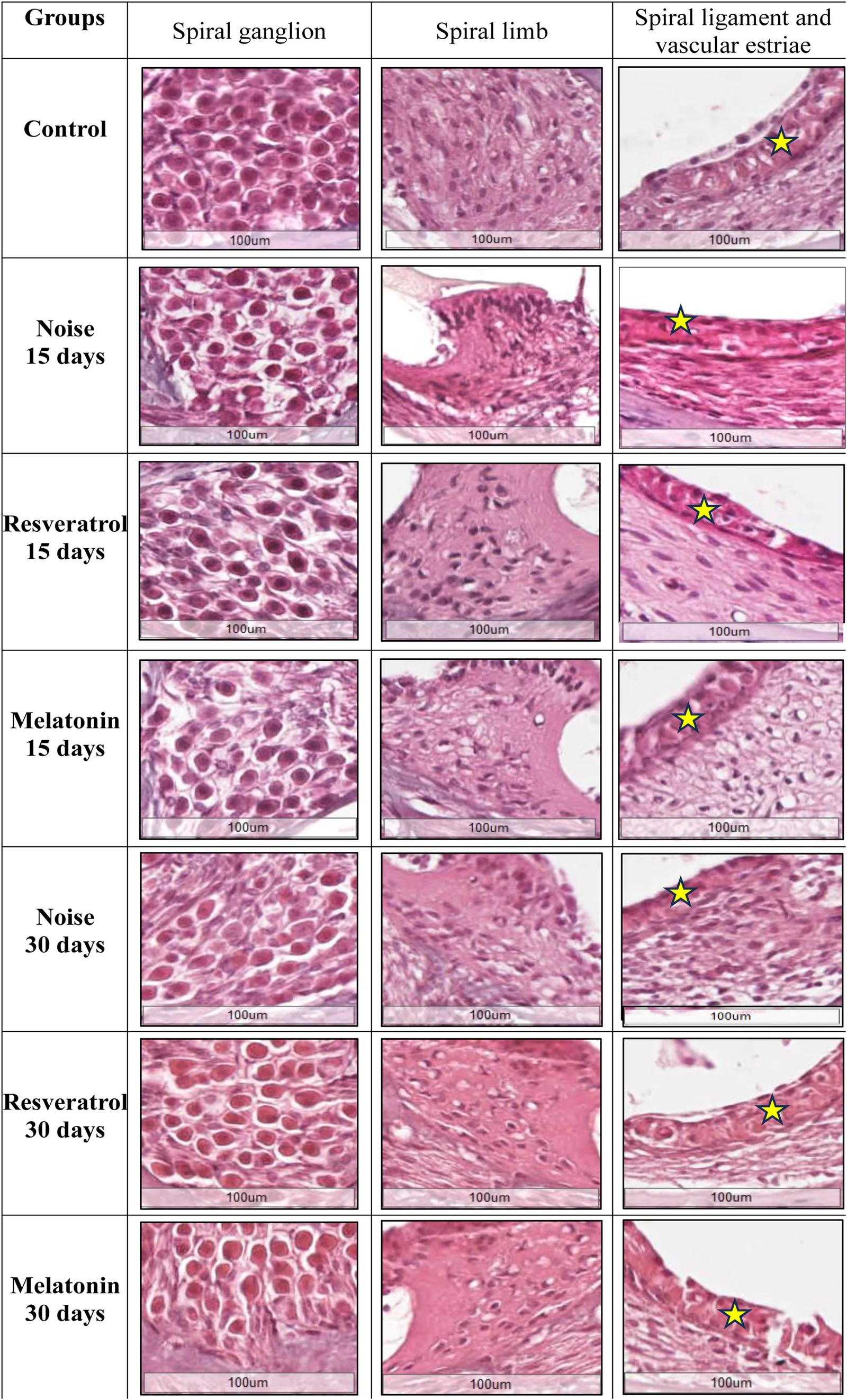
Representative photomicrographs of the results obtained for cell quantification from cochlear tissues of Wistar rats: spiral ganglion (first columm), spiral limbus (second columm), spiral ligament and stria vascularis (third columm). The groups of animals were exposed to white noise for 15 or 30 days and/or treated with 10 mg/kg/day of resveratrol or melatonin for 15 or 30 days. The epithelium of the vascular streak is highlighted by the yellow star. Hematoxylin & Eosin staining. Magnification bar = 100 μm.

### Spiral limbus

The analysis of the median of total viable cells in the limbus found a difference between groups C and No on D15 (Kruskal-Wallis p = 0.0019). The analysis performed on D30 found a statistical difference between group C and the exposed groups, Me/No, Re/No, and No (ANOVA p < 0.0001) (Fig. 1).

Fig. 2 shows a photomicrograph of the limbus of animals from group C and the noise-exposed groups, Me/No, Re/No, and No, after 10 days of noise exposure (D15). A higher cell density is observed in the group that was not exposed to noise. However, no statistical difference was observed between groups C and Me/No or Re/No.

### Spiral ligament

The animals in group C were statistically different in all analyses performed on D15 and D30 from the groups exposed to noise (ANOVA: p = 0.0006 on D15; p = 0.0001 on D30). On D15, although all exposed groups performed worse than group C, the No group was statistically different from the groups treated with resveratrol (Re/No) and melatonin (Me/No). On D30, this difference was maintained only between the No and Re/No groups (ANOVA: p = 0.0001) (Fig. 1).

### Stria vascularis

The analyses performed on D15 and D30 found a statistical difference between the C and the noise-exposed groups (ANOVA p < 0.0001). However, among the noise-exposed groups, the D15 analysis found that the Re/No group had less damage than the No group, with a statistical difference (ANOVA p = 0.001). This effect was not maintained in the evaluation on D30 (Fig. 1).

Fig. 2 shows the photomicrographs of the evaluated structures of the animals in the C and noise-exposed groups.

Data regarding the functional auditory analysis of this experimental model, including Distortion-Product Otoacoustic Emissions (DPOAE) and Auditory Brainstem Response (ABR) measurements, were previously published.^15^ This analysis demonstrated that exposure to white noise (100 dB SPL) caused significant cochlear damage, evidenced by reduced DPOAE amplitudes and elevated ABR electrophysiological thresholds. However, animals treated with melatonin and resveratrol showed relevant recovery of these parameters by D30, with ABR thresholds returning to levels statistically similar to those of the control group.

## Discussion

This study administered two antioxidants to mitigate the effects of noise exposure and conducted serial assessments on D15 and D30. The animals received the antioxidants for 4 days before the onset of noise exposure and throughout the exposure (10 days). It was decided to initiate the drug administration earlier to allow the potent antioxidant and anti-inflammatory properties to act preventively.

Synaptic disconnection between inner hair cells and type I neurons of the inner hair cell is a common early change in various forms of hearing loss, such as NIHL, ototoxicity, and aging. Glutamate excitotoxicity is believed to trigger this process, potentiated by oxidative stress generated by free radicals and nitric oxide, intensifying cochlear damage.^16^

The use of these antioxidants in mitigating glutamate-induced excitotoxicity has been previously reported. A study with melatonin^17^ demonstrated the reduction of oxidative stress and maintenance of mitochondrial membrane potential, in addition to modulating intracellular calcium influx and inhibiting MAPK phosphorylation, reinforcing its neuroprotective effects in retinal ganglion cells. Resveratrol also demonstrated its effects in reducing glutamate-induced damage, increasing the number of dopaminergic neurons, and improving their morphology in a study with primary mouse mesencephalic cultures.^18^

In the present study, the histological preservation of spiral ganglion neurons observed at D30 in the antioxidant-treated groups provides a structural basis that directly correlates with previously published functional findings.^15^ That previous study demonstrated that Wistar rats subjected to the same experimental protocol and treated with melatonin or resveratrol achieved complete recovery of ABR electrophysiological thresholds under click stimuli by D30, showing no statistical difference from the unexposed control group. Since spiral ganglion neurons are primarily responsible for the generation of auditory nerve action potentials captured in ABR wave complexes, the maintenance of cell density and morphological integrity observed here justifies the functional otoprotection reported in that literature. These combined data suggest that both compounds successfully mitigate glutamate-induced excitotoxicity and retrocochlear neural degeneration during the acoustic rest period.

Previous studies^19^^,^^20^ conducted in our laboratory had already demonstrated the beneficial action of melatonin on spiral ganglion neurons in animal models not exposed to noise. In the C57BL/6 J mouse model,^19^ melatonin had superior results in preventing age-related hearing loss, with a higher neuron count in the spiral ganglion. In a study on cisplatin-induced ototoxicity, animals treated with melatonin had a significantly higher cell density (6.8 cells/mm^2^) than the control group (1.7 cells/mm^2^).^20^ These findings, combined with the results of the present study, reinforce the role of melatonin in reducing oxidative stress, neutralizing the action of free radicals, and reinforcing its effectiveness in hearing protection.

Similarly, resveratrol was also cited for its otoprotective action in age-related hearing impairment by Yang et al.^21^ This effect is related to its inhibition of the RIPK1/RIPK3/MLKL-mediated necroptosis pathway, whose activation contributes to cochlear degeneration during aging. Histological analysis of organ of Corti cells and spiral ganglion neurons in aged mice showed a significant increase in the expression of these proteins in the cochlea. Administration of resveratrol at a dose of 50 mg/kg resulted in a significant reduction in the levels of RIPK1, RIPK3, and MLKL, demonstrating its inhibitory effect on necroptosis and suggesting a protective effect on cochlear structures.

Saidia et al.^16^ demonstrated that resveratrol also acts against excitotoxicity-induced cochlear synaptopathy. In an experimental model with cochlear explants, co-administration of resveratrol (10 μM) with Kainate (KA) prevented synapse loss between inner hair cells and spiral ganglion neurons, maintaining the number of synapses similar to that of unexposed controls. Furthermore, resveratrol treatment after KA exposure also significantly restored synaptic integrity, reinforcing its therapeutic potential.

In addition to the spiral ganglion, the structures of the spiral limbus, stria vascularis, and spiral ligament were analyzed. The effects of antioxidants were not as evident in these regions as those observed in the spiral ganglion. The stria vascularis is responsible for generating the endocochlear potential and maintaining high potassium (K^+^) concentrations in the endolymph, processes that occur in distinct compartments of the stria. The spiral limbus also participates in K^+^ regulation.^22^ K^+^ transport activity at these sites depends on the Na^+^/K^+^-ATPase present in stria and limbal cells. Noise exposure can overload these cells, compromise ion flux, and favor K^+^ accumulation along the proposed medial and lateral “recycling” pathways that guide K + from the organ of Corti back to the scala media.^22^^,^^23^

The results of the noise-exposed group without antioxidant treatment were similar to those observed by Ohlemiller and Gagnon,^24^ who reported acute cellular changes in the stria vascularis and spiral ligament in CBA mice exposed to broadband noise (110 dB SPL, 4–45 kHz, for 2 h). Eight weeks after exposure, the animals showed significant degeneration of the lateral wall of the cochlea and the central region of the limbus. In the present study, even after 15 days of auditory rest, the statistical difference in cell counts of the limbus, spiral ligament, and stria vascularis was maintained between the noise-exposed and control animals, including those treated with antioxidants.

However, the analysis performed on D15 indicated that animals exposed to noise and treated with resveratrol had less cell loss in the stria vascularis and spiral ligament than the untreated group, with statistically significant differences in the stria vascularis (D15) and spiral ligament (D30). These findings are corroborated by the study by Hanci et al.,^25^ who used electron microscopy and observed less cochlear damage in animals treated with resveratrol than in the untreated group.

Even though this study did not directly assess oxidative stress or inflammation levels, the histopathological findings complement previous evidence on the otoprotective effects of resveratrol. Seidman et al.^26^^,^^27^ demonstrated that cochlear hypoperfusion induces oxidative stress and the formation of Reactive Oxygen Species (ROS), compromising outer hair cell function. In these models, resveratrol reduced the expression of the inflammatory enzyme COX-2 and the production of ROS, indicating antioxidant and anti-inflammatory activity.

Regarding the lateral wall structures, the acute protection observed at D15 in the stria vascularis, and spiral ligament of animals treated with resveratrol is consistent with the partial functional recovery of outer hair cells detected in the earlier study.^17^ It was previously reported that although DPOAE amplitudes and Signal-to-Noise Ratios (SNR) significantly improved in the resveratrol and melatonin groups at D30 compared to the untreated noise group, their final values remained lower than those of the controls. This partial functional rescue aligns with the current histopathological findings, which showed that despite a transient reduction in cellular damage immediately after noise exposure (D15), a statistical difference in cell counts persisted between the antioxidant groups and the control group at D30. Because the stria vascularis and spiral ligament are crucial for maintaining the endocochlear potential and driving potassium recycling to sustain outer hair cell function, this histological evidence explains why the functional otoprotection captured by DPOAE was meaningful yet incomplete.

Finally, this study has some limitations that should be considered. The small number of animals per group may have limited the statistical power of the analyses, especially in the histological comparisons. Furthermore, the lack of electron microscopy prevented detailed ultrastructural observation of the cochlear cells, which could have complemented the morphological findings. Another limiting factor was the failure to include specific markers of oxidative stress, which would have allowed for better characterization of the cellular mechanisms involved in the action of antioxidants. Despite these limitations, the results provide relevant evidence on the otoprotective effects of melatonin and resveratrol and contribute to the advancement of knowledge in this field.

## Conclusion

Melatonin and resveratrol demonstrated a significant capacity for cochlear morphological preservation against noise exposure, particularly in sustaining spiral ganglion density, which underlies the functional otoprotection previously reported. Furthermore, resveratrol showed a distinct histological advantage in mitigating acute damage to the stria vascularis and spiral ligament.

## ORCID ID

Gabriela Guenther Ribeiro Novanta: 0000-0003-4494-3353

Ana Carolina Odorizzi Zica: 0000-0001-9671-4988

Maria Luiza Queiroz Sampaio: 0000-0002-9754-3786

Andressa Alves Caram: 0000-0001-6758-300X

Selma Aparecida Souza Kückelhaus: 0000-0001-7677-772X

Lucieny Silva Martins Serra: 0000-0002-8113-7222

Andre Luiz Lopes Sampaio: 0000-0001-7611-1303

## Authors’ contributions

Gabriela Guenther Ribeiro Novanta participated in the study design, data collection, analysis, discussion and writing of the manuscript; Ana Carolina Odorizzi Zica and Lucieny Silva Martins Serra participated in the study design, data collection, analysis, discussion, writing, and critical review of the manuscript; Maria Luiza Sampaio e Andressa Caram participated in the data collection, analysis, discussion and critical review of the manuscript; Selma Aparecida Souza Kückelhaus participated in the data collection, discussion and critical review of the manuscript; and Andre Luiz Lopes Sampaio participated in the study design, discussion, writing, and critical review of the manuscript.

## Funding

This research was funded by the Federal District Research Support Foundation – FAPDF.

## Data availability statement

The authors declare that all data are available in repository.

## Declaration of competing interest

The authors declare no conflicts of interest.
